# Absence of Functional Na_v_1.8 Channels in Non-diseased Atrial and Ventricular Cardiomyocytes

**DOI:** 10.1007/s10557-019-06925-6

**Published:** 2020-01-08

**Authors:** Simona Casini, Gerard A. Marchal, Makiri Kawasaki, Fransisca A. Nariswari, Vincent Portero, Nicoline W.E. van den Berg, Kaomei Guan, Antoine H.G. Driessen, Marieke W. Veldkamp, Isabella Mengarelli, Joris R. de Groot, Arie O. Verkerk, Carol Ann Remme

**Affiliations:** 1 Department of Experimental Cardiology, Amsterdam UMC, Meibergdreef 15, Amsterdam, 1105 AZ The Netherlands; 2 Institute of Pharmacology and Toxicology, Technische Universität Dresden, Fetscherstrasse 74, 01307 Dresden, Germany; 3 Department of Cardiology, Amsterdam UMC, Meibergdreef 15, Amsterdam, 1105 AZ The Netherlands; 4 Department of Medical Biology, Amsterdam UMC, Meibergdreef 15, Amsterdam, 1105 AZ The Netherlands

**Keywords:** *SCN10A*/Na_v_1.8, Sodium channel, Patch-clamp, Cardiomyocytes, Late sodium current, hiPSC-CMs

## Abstract

**Purpose:**

Several studies have indicated a potential role for *SCN10A*/Na_V_1.8 in modulating cardiac electrophysiology and arrhythmia susceptibility. However, by which mechanism *SCN10A*/Na_V_1.8 impacts on cardiac electrical function is still a matter of debate. To address this, we here investigated the functional relevance of Na_V_1.8 in atrial and ventricular cardiomyocytes (CMs), focusing on the contribution of Na_V_1.8 to the peak and late sodium current (*I*_Na_) under normal conditions in different species.

**Methods:**

The effects of the Na_V_1.8 blocker A-803467 were investigated through patch-clamp analysis in freshly isolated rabbit left ventricular CMs, human left atrial CMs and human-induced pluripotent stem cell-derived CMs (hiPSC-CMs).

**Results:**

A-803467 treatment caused a slight shortening of the action potential duration (APD) in rabbit CMs and hiPSC-CMs, while it had no effect on APD in human atrial cells. Resting membrane potential, action potential (AP) amplitude, and AP upstroke velocity were unaffected by A-803467 application. Similarly, *I*_Na_ density was unchanged after exposure to A-803467 and Na_V_1.8-based late *I*_Na_ was undetectable in all cell types analysed. Finally, low to absent expression levels of *SCN10A* were observed in human atrial tissue, rabbit ventricular tissue and hiPSC-CMs.

**Conclusion:**

We here demonstrate the absence of functional Na_V_1.8 channels in non-diseased atrial and ventricular CMs. Hence, the association of *SCN10A* variants with cardiac electrophysiology observed in, e.g. genome wide association studies, is likely the result of indirect effects on *SCN5A* expression and/or Na_V_1.8 activity in cell types other than CMs.

**Electronic supplementary material:**

The online version of this article (10.1007/s10557-019-06925-6) contains supplementary material, which is available to authorized users.

## Introduction

Sodium channels play a central role in the initiation and propagation of the action potential (AP) in excitable cells, including cardiomyocytes (CMs) and neurons***.*** Cardiac sodium channel (Na_V_1.5) loss of function is a critical mediator of cardiac conduction slowing, predisposing to ventricular arrhythmias and sudden cardiac death (SCD), both in acquired pathologies (ischemia, hypertrophy, heart failure) and inherited cardiac disorders caused by mutations in *SCN5A*, the gene encoding Na_V_1.5 [1, 2]. On the other hand, impaired Na_V_1.5 inactivation may induce a small inward sodium current (*I*_Na_), the so-called late *I*_Na_, that persists during the plateau and repolarization phase of the AP. Enhanced late *I*_Na_ can prolong AP duration (APD) and increase intracellular calcium (Ca^2+^) via altered Na^+^/Ca^2+^ exchanger activity, thus promoting arrhythmias [3]. While Na_V_1.5 is the main sodium channel isoform expressed in cardiac tissue, other sodium channel isoforms are also present in the heart, including Na_V_1.1/*SCN1A*, Na_V_1.2/*SCN2A*, Na_V_1.3/*SCN3A* and Na_V_1.6/*SCN8A* [4]. These isoforms are typically referred to as “neuronal” sodium channel isoforms due to their abundant expression and well-established function in neurons. While Na_V_1.5 is blocked only by micromolar concentrations of tetrodotoxin (TTX) [i.e. TTX-resistant], most neuronal isoforms are more TTX-sensitive and inhibited by nanomolar concentrations [4]. An exception is Na_V_1.8, encoded by the *SCN10A* gene. This isoform is mainly expressed in dorsal root ganglia, plays a role in pain perception [5] and is inhibited only by micromolar TTX concentrations, similar to Na_V_1.5 [4].

Several genome-wide association studies (GWAS) have suggested a role for *SCN10A*/Na_V_1.8 in modulating cardiac conduction parameters, such as PR and QRS interval [6–10]. *SCN10A* variants have also been associated with atrial fibrillation (AF) [11–13] and with Brugada syndrome [14–16], an inherited cardiac disease characterized by cardiac conduction slowing and increased risk for SCD. However, if and by which mechanism *SCN10A*/Na_V_1.8 impacts on cardiac electrical function is still a matter of debate. Inhibition of Na_V_1.8 by the blocker A-803467 has been reported to decrease late *I*_Na_ and shorten APD in mouse and rabbit cardiomyocytes [17], whereas we previously described the absence of functional Na_V_1.8 in murine cardiomyocytes [18]. Moreover, conflicting results have been reported in studies of mice deficient for *Scn10a*, with either similar or decreased APD observed in *Scn10a*^−/−^ cardiomyocytes as compared to wild-type cardiomyocytes [17, 19]. We and others have shown that Na_V_1.8 is specifically expressed in murine, canine and human cardiac neurons [18, 20, 21], suggesting a function of the *SCN10A* gene product for cardiac conduction via modulation of AP firing in intracardiac neurons [18, 21, 22]. Additionally, the *SCN10A* variant rs6801957 has been shown to modulate *SCN5A* expression in cardiac tissue thereby potentially impacting on conduction [23]. Overall, the role of *SCN10A*/Na_V_1.8 in the heart and the mechanisms by which this gene and/or its gene product affects cardiac function remain only partially explained. In particular, electrophysiological studies in non-diseased human cardiomyocytes aimed at defining the physiological role of Na_V_1.8 in the human heart are limited. To address these issues, we here investigated the functional relevance of Na_V_1.8 in atrial and ventricular cardiomyocytes, focusing on the contribution of Na_V_1.8 to the peak and late *I*_Na_ under normal conditions in different species.

## Methods

### Isolation of Rabbit Left Ventricular Cardiomyocytes

Three–four-month-old male New Zealand White rabbits (Charles River Laboratories) were anaesthetized with 20 mg xylazine and 100 mg ketamine (intramuscularly) and heparinized with a bolus of 1000 IU heparin (intravenously). Subsequently, the animals were sacrificed, the thorax was opened and the heart was rapidly excised and connected to a Langendorff system. Left ventricular (LV) cardiomyocytes were isolated as previously described [24] (see Data Supplement).

### Isolation of Human Left Atrial Cardiomyocytes

Human left atrial appendages (LAAs) were obtained from patients in sinus rhythm (SR) without a history of AF undergoing cardiac surgery (coronary bypass grafting or valve surgery) and included in the multicenter PREDICT AF study [25]. Patient characteristics are reported in Supplemental Table 1. Part of the LAA tissue was immediately frozen in liquid nitrogen to be subsequently used for molecular analysis, while the other part was transported to the laboratory on ice and single cells were obtained by an enzymatic isolation modified from Dobrev et al. [26]. An expanded Methods section is available in the Data Supplement.

### Differentiation of hiPSCs into Cardiomyocytes

A human-induced pluripotent stem cell (hiPSC) control line (iC113) previously generated and characterized [27] was used to generate cardiomyocytes (hiPSC-CMs) by adaptation of a previously described protocol [28]. hiPSC-CMs were used for electrophysiological analysis and RT-PCR. An expanded Methods section is available in the Data Supplement.

### Electrophysiology

#### Data Acquisition and Analysis

Membrane currents [(*I*_Na_, late *I*_Na_ and L-type calcium currents (*I*_CaL_)] and APs were measured with the ruptured and perforated patch-clamp technique, respectively, using an Axopatch 200B amplifier (Molecular Devices, San Jose, CA, USA). Voltage control, data acquisition and analysis of currents and APs were performed with pClamp10.6/Clampfit (Molecular Devices, San Jose, CA, USA) or a custom-made software. Borosilicate glass patch pipettes (Harvard Apparatus, Holliston, MA, USA) with a tip resistance of 2–2.5 MΩ were used. Series resistance (Rs) and cell membrane capacitance (Cm) were compensated for 80%. Peak *I*_Na_, *I*_CaL_ and APs were filtered at 5 kHz. *I*_Na_ and APs were digitized at 40 kHz, while *I*_CaL_ was digitized at 20 kHz. Finally, late *I*_Na_ was filtered and digitized at 2 kHz and 1 kHz, respectively.

#### Sodium Current Measurements

Peak *I*_Na_ and late *I*_Na_ were measured in single cells using a pipette solution containing (in mM) 3.0 NaCl, 133 CsCl, 2.0 MgCl_2_, 2.0 Na_2_ATP, 2.0 TEACl, 10 EGTA, 5.0 HEPES; pH 7.2 (CsOH). For late *I*_Na_ measurements, hiPSC-CMs, rabbit and human CMs were superfused with a bath solution containing (in mM) 130 NaCl, 10 CsCl, 1.8 CaCl_2_, 1.2 MgCl_2_, 11.0 glucose, 5.0 HEPES, 0.005 nifedipine; pH 7.4 (CsOH). For peak *I*_Na_ recordings, a similar bath solution was used with the exception of a lower NaCl concentration for proper voltage control. Hence, NaCl was replaced by CsCl (for rabbit and human CMs, we used 7 mM NaCl and 133 mM CsCl; for hiPSC-CMs: 20 mM NaCl and 120 mM CsCl). Peak *I*_Na_ was measured at room temperature in response to depolarizing voltage steps from a holding potential of − 120 mV (cycle length of 5 s). *I*_Na_ was defined as the difference between peak and steady-state current (at 500 ms). Voltage dependence of activation and inactivation curves was fitted with Boltzmann function (*y* = [1 + exp.{(*V-V*_*1/2*_)/*k*}]^−1^), where *V*_*1/2*_ is the half-maximal voltage of (in) activation and *k*, the slope factor*.* Na_v_1.8-based late *I*_Na_ and total late *I*_Na_ were measured at 36 °C, as A-803467 (100 nM) and TTX (30 μM)-sensitive currents, respectively, using descending ramp protocols (cycle length of 5 s) as depicted in Fig. 2 and Supplemental Fig. 4. Current densities were calculated by dividing current amplitude by Cm. Cm was determined by dividing the decay time constant of the capacitive transient in response to 5 mV hyperpolarizing steps from − 40 mV, by the Rs. Potentials for peak *I*_Na_ and late *I*_Na_ recordings were not corrected for the estimated change in liquid junction potential. The Na_V_1.8 channel inhibitor A-803467 (Tocris Bioscience, Bristol, United Kingdom) was solubilized in DMSO at a stock solution of 10 mM and diluted to the final concentration of 100 nM before use. This dose was chosen based on previous IC_50_ data and to ensure maximal inhibition of Na_v_1.8-based current [29].

#### L-Type Calcium Current Measurements

*I*_CaL_ was measured in isolated rabbit left ventricular CMs at 36 °C. An expanded Methods section is available in the Data Supplement.

#### Action Potential Measurements

In single rabbit left ventricular CMs, human left atrial (LA) CMs and hiPSC-CMs, APs were measured at 36 °C using a modified Tyrode’s solution containing (in mM) 140 NaCl, 5.4 KCl, 1.8 CaCl_2_, 1.0 MgCl_2_, 5.5 glucose, 5 HEPES, pH 7.4 (NaOH). Pipettes were filled with (in mM) 125 K-gluconate, 20 KCl, 5 NaCl, 0.44 amphotericin-B, 10 HEPES, pH 7.2 (KOH). APs were elicited at 1 Hz by 3 ms, ≈ 1.2× threshold current pulses through the patch pipette. Typically, hiPSC-CMs have a small or even complete lack of the inward rectifying potassium current (*I*_K1_). Consequently, their resting membrane potential (RMP) is depolarized and they are frequently spontaneously active [30]. To overcome these conditions, which limit the functional availability of *I*_Na_, transient outward potassium current and L-type Ca^2+^ current [31], we injected an in silico *I*_K1_ with kinetics of Kir2.1 channels through dynamic clamp [32]. Thus, cells became quiescent with a RMP of around − 80 mV. We analysed RMP, AP amplitude (APA), maximal AP upstroke velocity (*V*_max_) and AP duration (APD) at 50% and 90% repolarization (APD_50_ and APD_90_, respectively). Data from 10 consecutive APs were averaged and potentials were corrected for the calculated liquid junction potential of 15 mV [33].

## Real-Time Polymerase Chain Reactions

Total RNA was isolated from left atrial appendages of five patients undergoing cardiac surgeries using TRIzol Reagent (Invitrogen, Waltham, MA, USA) and from hiPSC-CMs obtained from four independent differentiations using NucleoSpin RNA (MACHEREY-NAGEL ref.: 740955.50, Duren, Germany) following manufacture protocol. cDNA was synthesized from total RNA by SuperScript™ II Reverse Transcriptase (Invitrogen, Waltham, MA, USA). Real-time PCR was performed on the platform of Light Cycler 480 (Roche, Basel, Switzerland) using SYBR green I master mix (Roche, Basel, Switzerland) and the sets of primers reported in Supplemental Table 2. Gene expression was determined according to linear regression analysis using LinRegPCR software and normalized by the expression of hypoxanthine phosphoribosyltransferase (*HPRT).*

## RNA Sequencing Data Analysis

*SCN5A* and *SCN10A* expression in human right and left atria [34], ventricular and atrial hiPSC-CMs [35] and rabbit left ventricular tissue were extrapolated from the RNA sequencing (RNA-Seq) datasets GSE31999, GSE111007 and GSE115605, respectively, which are publicly available online https://www.ncbi.nlm.nih.gov/geo/. For the analyses, read counts for *SCN5A* and *SCN10A* transcripts were normalized to millions of total reads generated per sample and to *SCN5A* (ENST00000413689.1) and *SCN10A* (ENST00000449082.2) transcript size (i.e. Fragment Per Kilobase Million, FPKM).

## Statistical Analysis

Values are shown as mean ± SEM. Paired Student’s *t* test, unpaired Student’s *t* test, one-way repeated measures ANOVA followed by Holm-Sidak test for post hoc analyses and two-way repeated measures ANOVA were used when appropriate. Mann-Whitney *U* test and one-way repeated measures ANOVA on Ranks (Friedman test) followed by Tukey test for post hoc analyses were used for data not normally distributed. The level of statistical significance was set to *p* < 0.05.

## Results

### Effect of A-803467 on AP Properties in Atrial and Ventricular Cardiomyocytes

**Fig. 1:**
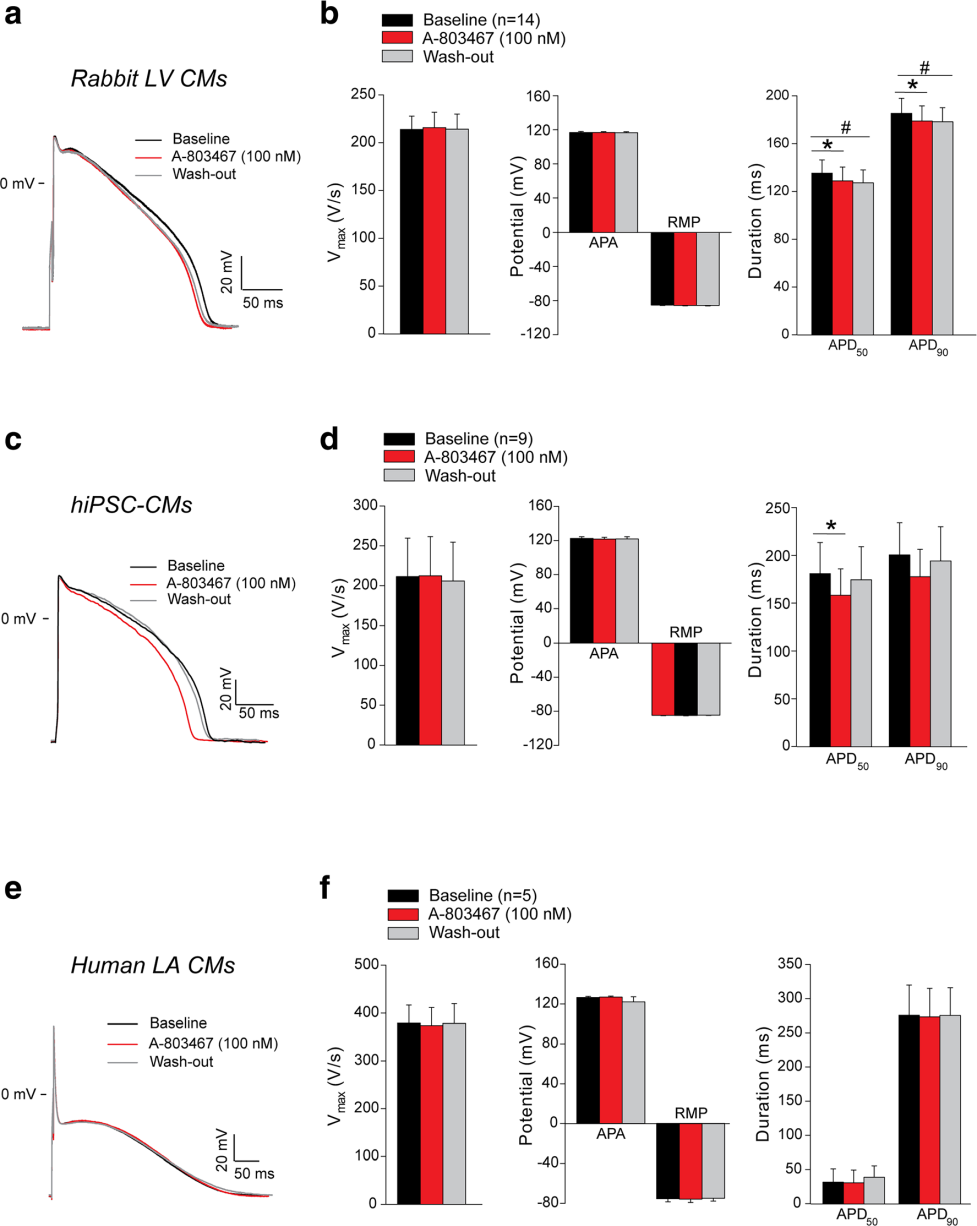
Effect of A-803467 treatment on action potential (AP) properties in ventricular and atrial cardiomyocytes. **a**, **c**, **e** Examples of APs recorded at the stimulation frequency of 1 Hz in rabbit left ventricular (LV) cardiomyocytes (CMs) (**a**), human-induced pluripotent stem cell-derived CMs (hiPSC-CMs) (**c**) and human left atrial (LA) CMs (**e**) under physiological conditions (baseline), after 5-min wash-in of 100 nM A-803467 and after 5-min wash-out of the drug. **b**, **d**, **f** Average data at 1 Hz for maximal upstroke velocity (*V*_max_), AP amplitude (APA), resting membrane potential (RMP), AP duration at 50% and 90% repolarization (APD_50_ and APD_90_), before (baseline) and after wash-in and wash-out of 100 nM A-803467 in rabbit LV CMs, hiPSC-CMs and human LA CMs. *p < 0.05 baseline vs A-803467, ^#^*p* < 0.05 baseline vs wash-out; one-way repeated measures ANOVA followed by Holm-Sidak test for post hoc analyses or one-way repeated measures ANOVA on Ranks (Friedman test)  followed by Tukey test for post hoc analyses when data were not normally distributed

We first assessed the effects of the Na_V_1.8 blocker A-803467 on AP properties. Figure 1a, c, e shows typical AP recordings obtained from rabbit left ventricular CMs, hiPSC-CMs and human left atrial CMs under physiological conditions (baseline), in the presence of 100 nM A-803467 and after wash-out of the drug. On average, maximal upstroke velocity, AP amplitude and resting membrane potential were not affected by A-803467 exposure (Fig. 1b, d, f, Supplemental Table 3). In rabbit ventricular CMs, we observed a small, yet significant, APD shortening induced by A-803467 treatment (APD reduction of 4.8% for APD_50_ and of 3.5% for APD_90_, Fig. 1b, Supplemental Table 3). However, as shown in detail in Supplemental Fig. 1a and b, the observed APD reduction was (partly) reversible upon wash-out only in a minority of cells. In the majority of cases, either A-803467 did not affect APD or the effect was non-reversible. Moreover, time-matched control experiments in rabbit ventricular CMs showed APD_50_ and APD_90_ shortening occurring over time similar to that observed with A-803467 (Supplemental Figs. 2 and 3). In human left atrial CMs, exposure to A-803467 did not change APD (Fig. 1e, f, Supplemental Fig. 1e, f and Supplemental Table 3), and in hiPSC-CMs, only APD_50_ was significantly reduced but not APD_90_ (Fig. 1c, d, Supplemental Table 3). The effect of A-803467 was reversible upon wash-out in the majority of hiPSC-CMs, but the blocker did not affect APD in all cells (Supplemental Fig. 1c and d).

## Absence of Na_V_1.8-Based Late *I*_Na_ in Ventricular and Atrial Cardiomyocytes

**Fig. 2:**
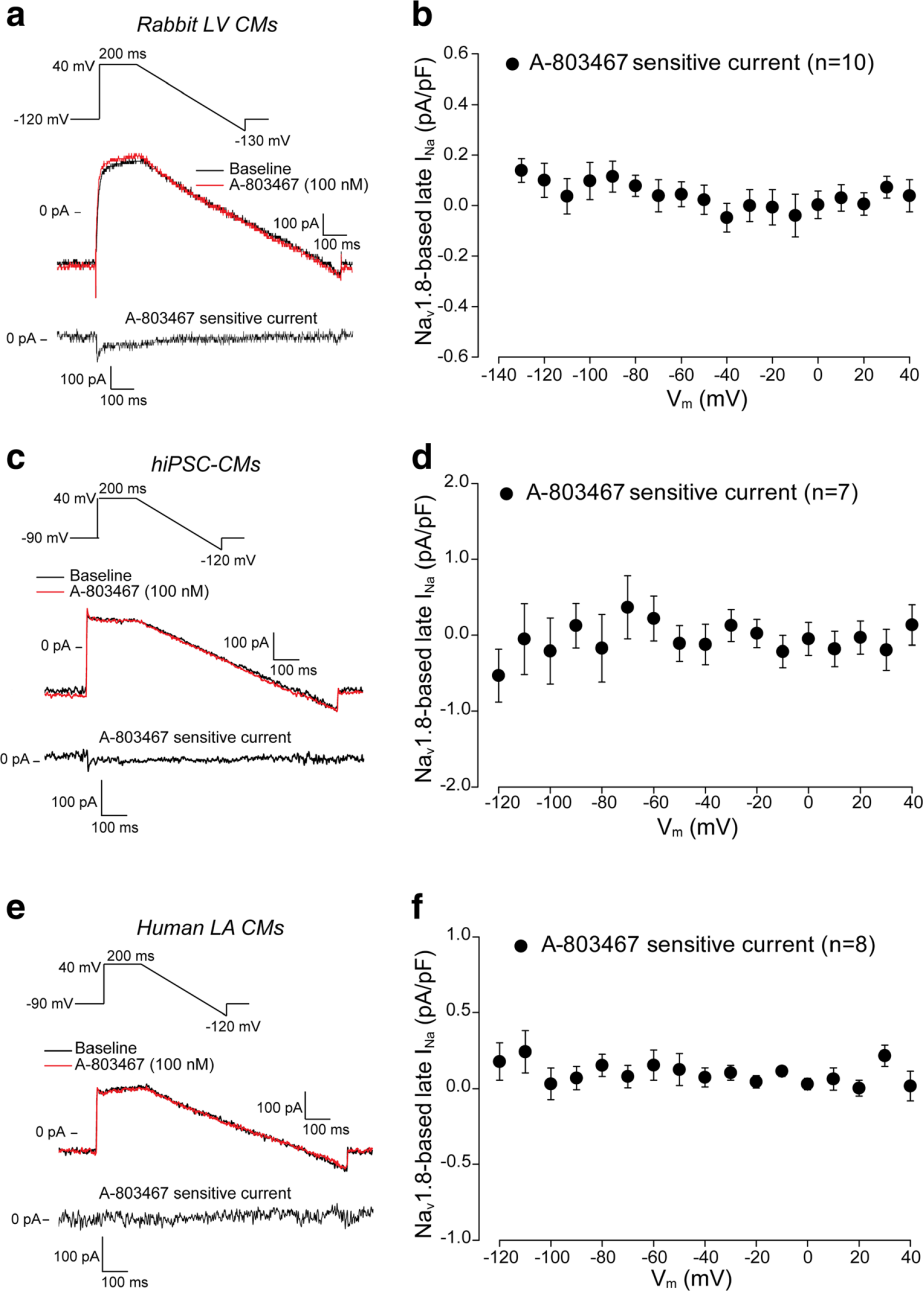
Absence of Na_V_1.8-based late sodium current (*I*_Na_) in ventricular and atrial cardiomyocytes. **a**, **c**, **e** Representative Na_v_1.8-based late *I*_Na_ traces recorded during a ramp protocol (see insets) in rabbit left ventricular (LV) cardiomyocytes (CMs) (**a**), human-induced pluripotent stem cell-derived CMs (hiPSC-CMs) (**c**) and human left atrial (LA) CMs (**e**) at baseline and after 5-min application of 100 nM A-803467. A-803467 sensitive current was obtained by subtraction of the current recorded in the presence of A-803467 from the current recorded earlier in the absence of the compound. **b**, **d**, **f** Average current-voltage (I-V) relationships for Na_V_1.8**-**based late *I*_Na_ measured as A-803467 sensitive current in rabbit LV CMs (**b**), hiPSC-CMs (**d**) and human LA CMs (**f**)

We next investigated the effects of A-803467 on late *I*_Na_ in rabbit left ventricular CMs, hiPSC-CMs and human left atrial CMs using descending ramps after a 200 ms pre-pulse to 40 mV (see inset of Fig. 2a, c, e). The advantage of using a ramp protocol instead of a single step protocol is that the ramp protocol allows measurements of late *I*_Na_ across a dynamic voltage range simulating a plateau and repolarization phase of an AP [36]. Figure 2 a, c, and e show typical examples of Na_v_1.8-based late *I*_Na_ recordings under basal conditions (baseline, black line) and after 5 min wash-in of 100 nM A-803467 (red line). Na_V_1.8-dependent late *I*_Na_, measured as A-803467-sensitive current, was obtained by subtraction of the current recorded in the presence of A-803467 from the current recorded in the absence of the compound. Na_V_1.8-dependent late *I*_Na_ was not detected in any of the three cell types (Fig. 2b, d, f). In a subset of rabbit left ventricular CMs, A-803467 perfusion was followed by 30 μM TTX application, and total late *I*_Na_ was measured as TTX-sensitive current obtained by subtraction of the current recorded in the presence of TTX from the current recorded earlier in the absence of TTX (Supplemental Fig. 4). Average total late *I*_Na_ was around − 0.2 pA/pF, while the A-803467 sensitive current was undetectable. Hence, these experiments demonstrate that functional Na_V_1.8-based late *I*_Na_ is absent under basal conditions in atrial and ventricular cardiomyocytes.

## Absence of Na_V_1.8-Based Peak *I*_Na_ in Ventricular and Atrial Cardiomyocytes

**Fig. 3:**
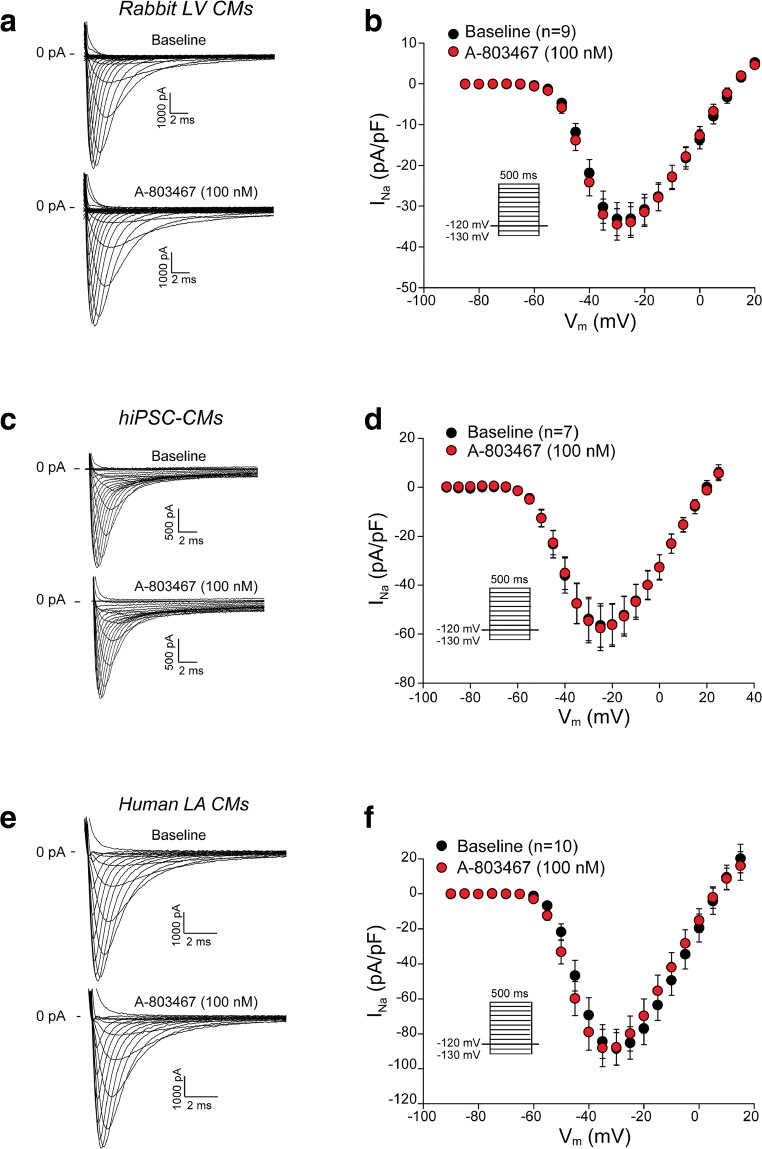
Na_v_1.8 does not contribute to peak sodium current (*I*_Na_) in ventricular and atrial cardiomyocytes. **a**, **c**, **e** Representative peak *I*_Na_ traces recorded from rabbit left ventricular (LV) cardiomyocytes (CMs) (**a**), human-induced pluripotent stem cell-derived CMs (hiPSC-CMs) (**c**) and human left atrial (LA) CMs (**e**) under physiological conditions (baseline) and after 5-min wash-in of 100 nM A-803467. **b**, **d**, **f** Average current-voltage (I-V) relationships at baseline and in the presence of 100 nM A-803467 in rabbit LV CMs (**b**), hiPSC-CMs (**d**) and human LA CMs (**f**). Insets: voltage protocols. Statistical test applied: two-way repeated measures ANOVA

In addition to late *I*_Na_, we also investigated the effects of A-803467 on peak *I*_Na_ density and voltage dependency of activation and inactivation. Figure 3a, c, e shows typical peak *I*_Na_ recordings obtained from rabbit left ventricular CMs, hiPSC-CMs and human left atrial CMs under basal conditions (baseline) and after 5 min wash-in of 100 nM A-803467. Average peak *I*_Na_ densities were unchanged after exposure to A-803467 in all cell types analysed (Fig. 3b, d, f, Supplemental Table 4).

**Fig. 4:**
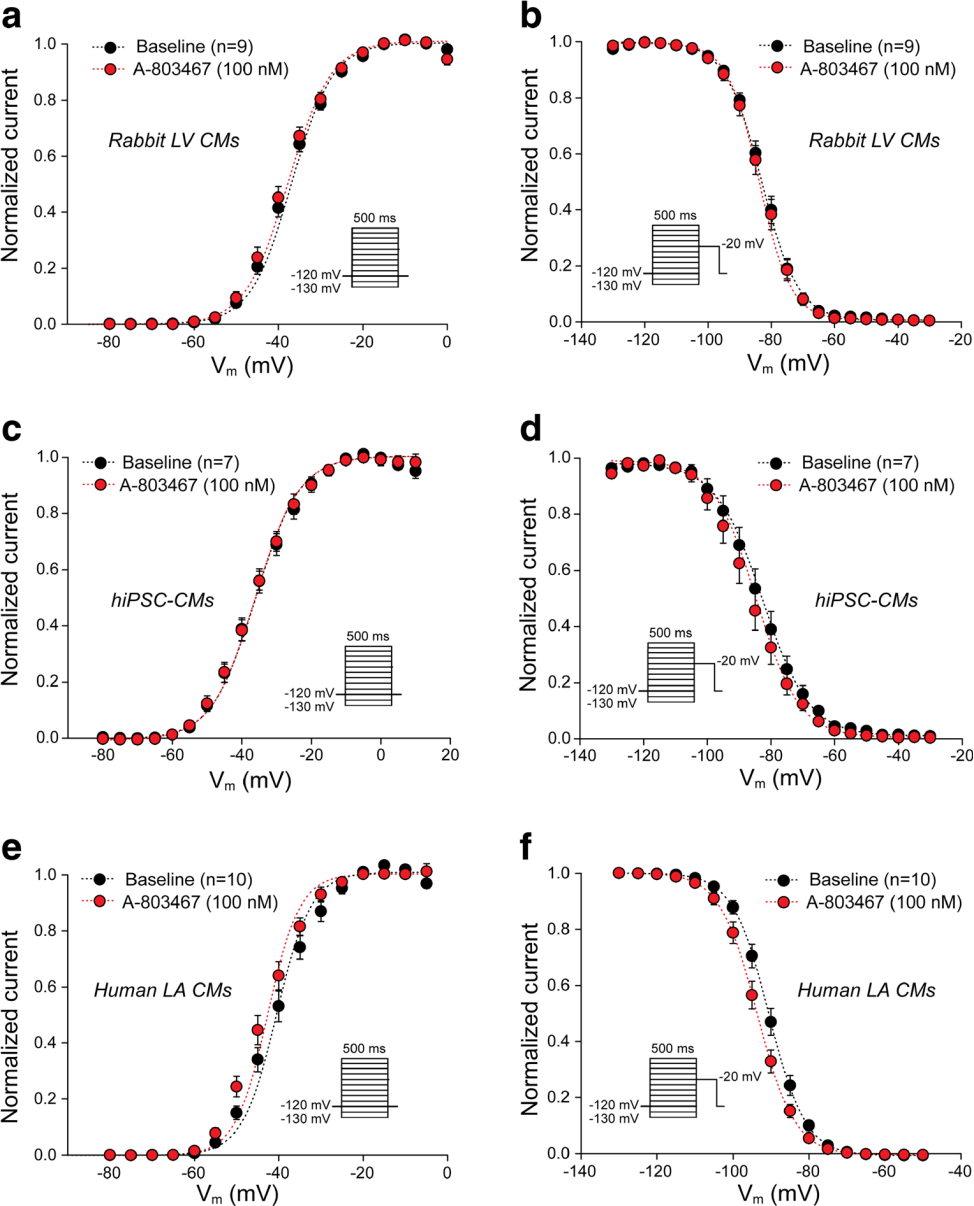
Effect of A-803467 treatment on sodium current (*I*_Na_) voltage dependence of activation and inactivation in ventricular and atrial cardiomyocytes. Average *I*_Na_ voltage dependence of activation (**a**, **c**, **e**) and inactivation (**b**, **d**, **f**) in rabbit left ventricular (LV) cardiomyocytes (CMs), human-induced pluripotent stem cell-derived CMs (hiPSC-CMs) and human left atrial (LA) CMs under basal conditions (baseline) and after 5-min exposure to 100 nM A-803467. Insets: voltage protocols. Statistical test applied: paired Student’s *t* test (see Supplemental Table 4)

*I*_Na_ voltage dependence of activation and inactivation, assessed as the half voltage of (in)activation (*V*_1/2_) and the slope factor *k*, was not affected by A-803467 in rabbit CMs and hiPSC-CMs (Fig. 4a–d, Supplemental Table 4). A-803467 caused a small negative shift in *V*_1/2_ of activation and inactivation (− 2.8 mV for both activation and inactivation curve) in human LA cardiomyocytes (*V*_1/2_ activation − 40.5 ± 1.1 mV vs − 43.3 ± 1.2 mV, *p* < 0.05, paired Student’s *t* test; *V*_1/2_ inactivation − 90.6 ± 0.9 mV vs − 93.4 ± 1.0 mV, *p* < 0.05, paired Student’s *t* test; Supplemental Table 4). Although significant, the biological meaning of such a small change is questionable. Moreover, in a subset of cells where wash-out experiments were also performed, we were unable to reverse these effects of A-803467, and a further negative shift of *V*_1/2_ of (in)activation was observed upon wash-out of the compound (Supplemental Fig. 5). These results suggest a time-dependent shift of (in)activation, rather than a A-803467-dependent effect on *I*_Na_ kinetics [37]. Taken together, these findings demonstrate the absence of functional Na_v_1.8-based peak *I*_Na_ in atrial and ventricular cardiomyocytes.

To explore a potential off-target effect of A-803467, we also measured its effects on the L-type calcium current (*I*_CaL_) in rabbit left ventricular CMs. Typical example of *I*_CaL_ traces recorded under basal conditions (baseline) and in the presence of A-803467 are shown in Supplemental Fig. 6a. Exposure to A-803467 affected neither *I*_CaL_ density (Supplemental Fig. 6b, Supplemental Table 5) nor *I*_CaL_ voltage dependence of activation and inactivation (Supplemental Fig. 6c, Supplemental Table 5).

## Low *SCN10A* mRNA Transcript Levels in hiPSC-CMs and in Human Left Atrial Appendages

**Fig. 5:**
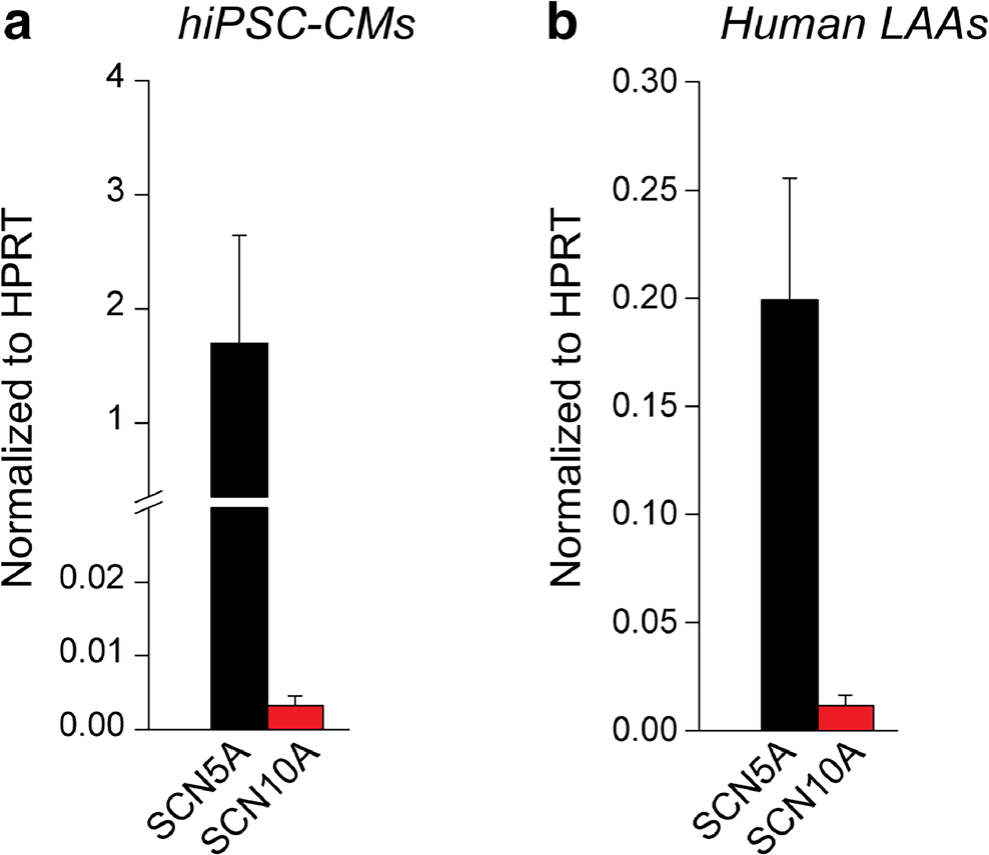
*SCN10A* and *SCN5A* expression levels in human-induced pluripotent stem cell-derived cardiomyocytes (hiPSC-CMs) and human left atrial tissue. *SCN5A* and *SCN10A* mRNA levels in hiPSC-CMs (**a**) and in human left atrial appendages (LAAs) (**b**). Gene expression was normalized to the reference gene *HPRT*

**Fig. 6:**
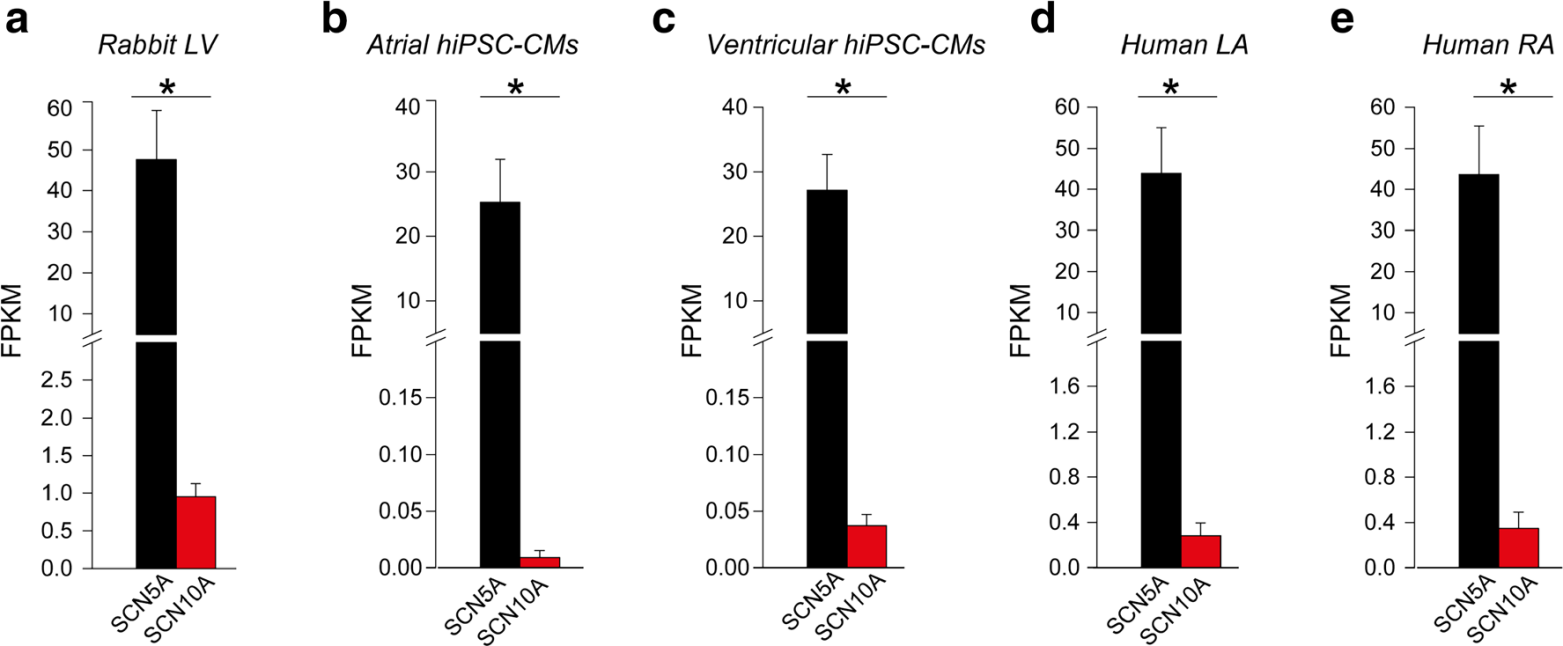
RNA sequencing (RNA-Seq) data analysis for *SCN5A* and *SCN10A* expression levels in rabbit left ventricle, induced pluripotent stem cell-derived cardiomyocytes (hiPSC-CMs) and human atria. *SCN5A* and *SCN10A* expression levels in rabbit left ventricle (LV) (**a**), atrial and ventricular hiPSC-CMs (**b**, **c)** and human left (LA) and right atria (RA) (**d**, **e**) were extrapolated from analysis of online RNA sequencing (RNA-Seq) raw datasets previously published [34, 35]. Read counts for *SCN5A* and *SCN10A* transcripts were normalized to millions of total reads generated per sample (six samples for rabbit left ventricular tissue and four samples for hiPSC-CMs and human atria) and to *SCN5A* and *SCN10A* transcript size (i.e. fragment per kilobase million, FPKM). *p < 0.05; unpaired Student’s *t* test or Mann-Whitney *U* test when data were not normally distributed

We finally assessed the mRNA expression levels of *SCN10A* in hiPSC-CMs and human LAAs using quantitative real-time PCR (RT-PCR). In LAAs tissue, RT-PCR was performed on the same samples used for AP and late *I*_Na_ measurements. As expected, both hiPSC-CMs and LAAs tissue showed robust expression of the cardiac sodium channel isoform *SCN5A* (relative to the reference gene *HPRT*). In contrast, *SCN10A* transcript levels were very low in both hiPSC-CMs and human LAAs (Fig. 5). Similarly, low to almost absent expression of *SCN10A* as compared to *SCN5A* was observed in online RNA-Seq datasets of rabbit left ventricular tissue (GSE115605) (Fig. 6a), atrial and ventricular hiPSC-CMs (GSE111007) (Fig. 6b, c) [35] and human left and right atria (GSE31999) (Fig. 6d, e) [34]. These observations are in line with our patch-clamp data showing the absence of functional Na_V_1.8-based sodium channels under basal conditions in atrial and ventricular CMs.

## Discussion

While several studies have previously implicated Na_V_1.8 in modulating cardiac electrophysiology and arrhythmia susceptibility, the underlying mechanism(s) are still a matter of debate. To address this, we here investigated the functional relevance of Na_V_1.8 in atrial and ventricular cardiomyocytes, focusing on the contribution of Na_V_1.8 to the peak and late *I*_Na_ under physiological conditions. Using detailed patch-clamp analyses of atrial and ventricular myocytes from different species, we observed a lack of effect of the Na_V_1.8 blocker A-803467 on peak and late *I*_Na_ in cardiomyocytes. In line with these observations, molecular investigation showed a virtual absence of *SCN10A* mRNA in human atrial tissue and hiPSC-CMs. Similarly, analysis of online RNA-Seq datasets of rabbit ventricular tissue, ventricular and atrial hiPSC-CMs and human right and left atria revealed low to almost absent expression of *SCN10A* as compared to *SCN5A*. Hence, our results demonstrate the absence of functional Na_V_1.8 channels in non-diseased atrial and ventricular cardiomyocytes, which is of particular relevance when extrapolating findings on *SCN10A* mutations and (common) variants.

Our findings are in contrast to the study of Yang et al. [17], which suggested that Na_V_1.8 is a component of late *I*_Na_ in non-diseased cardiomyocytes, and as such may modulate arrhythmia susceptibility [17]. Yang et al. showed that in mouse and rabbit ventricular cardiomyocytes, application of A-803467 reduced late *I*_Na_ and shortened APD, without affecting peak *I*_Na_ density [17]. In contrast, we did not detect any Na_V_1.8**-**based late *I*_Na_ in our cardiomyocytes. This discrepancy could be due to species differences (mouse vs human) and/or to different experimental conditions such as different temperature (room temperature [17] versus physiological temperature used by us). Indeed, in a recent study, Poulet et al. [38] reported a significant increase in late *I*_Na_ in human right atrial cardiomyocytes from AF patients as compared to patients in SR when experiments were conducted at room temperature. However, at physiological temperature, the difference in late *I*_Na_ amplitudes between SR and AF cells was less pronounced and did not reach the level of statistical significance [38]. In our study, A-803467 reduced APD_90_ by only 3.5% in rabbit ventricular CMs, while in the study of Yang et al. [17], the reduction was ~ 30%. Again, different experimental conditions may underlie these discrepancies, for instance the use of the perforated patch vs rupture patch, differences in recording solutions and temperature. Moreover, in the study of Yang et al., wash-out of A-803467 was not investigated, leaving the possibility that part of the observed effect was a time-dependent effect. Indeed, in time-matched AP recordings in rabbit cardiomyocytes, we observed an APD reduction over time similar to that observed with A-803467 suggesting that the apparent APD shortening induced by A-803467 in this species is a non-specific effect independent of the blocker. A similar small APD reduction was also observed in mouse ventricular CMs (but not in mouse atrial CMs) in our previous study [18]. Finally, despite the absence of Na_V_1.8-based peak and late *I*_Na_, we still observed a small yet significant and mostly reversible decrease in APD in hiPSC-CMs induced by A-803467. This AP shortening could be due to a potential off-target effect of A-803467. Although we observed no effects of A-803467 on *I*_CaL_ in rabbit, we cannot completely rule out effects of A-803467 on other ion channels, cautioning its use in electrophysiological studies aimed at establishing the functional relevance of Na_V_1.8 in, e.g. arrhythmogenesis.

A number of previous studies have suggested a role for *SCN10A*/Na_V_1.8 in modulating cardiac conduction and arrhythmogenesis. Perhaps the most compelling evidence on a potential role for Na_V_1.8 came from studies in mice deficient for *Scn10a* (*Scn10a*^−/−^). In ventricular CMs isolated from *Scn10a*^−/−^ mice, APs were shorter than those in wild-type mice and A-803467 had no effect on peak or late *I*_Na_, nor on APD, thus supporting the idea of a contribution of Na_V_1.8 to late *I*_Na_ [17]. However, in a follow-up study, the same authors reported similar APD in wild-type and *Scn10a*^−/−^ ventricular cardiomyocytes at baseline conditions, and only under extreme experimental conditions, e.g. after pre-treatment with the late *I*_Na_ enhancer ATX II, a reduced late *I*_Na_ was observed in knockout ventricular CMs [19]. Nevertheless, ATX-II administration in anesthetized mice and Langendorff-perfused hearts prolonged QTc and induced arrhythmias to the same extent in wild-type and *Scn10a*^−/−^ mice. Finally, no *Scn10a* transcript was detected in either wild-type or *Scn10a*^−/−^ ventricular CMs and ECG parameters were similar in both wild-type *Scn10a*^−/−^ mice [19], further underlining the limited relevance of Na_V_1.8 in cardiomyocytes under physiological conditions.

Mutations or variants in *SCN10A* (associated with both gain and loss of Na_v_1.8-based sodium channel function) have been associated with inherited arrhythmia syndromes such as Brugada syndrome [14–16], as well as increased AF susceptibility [11–13]. However, functional relevance of these identified mutations and variants has only been assessed in heterologous expression systems such as HEK-293 cells which differ significantly from the cardiomyocyte environment [10, 12, 13, 15, 16]. Nonetheless, even if a mutation in *SCN10A* is found to alter function of Na_V_1.8-based channels in expression systems, this does not automatically imply a functional impact on the cardiomyocyte level. In fact, our current and previous findings demonstrate very low expression levels of *SCN10A* and the consequent absence of functional Na_V_1.8-based current in atrial and ventricular CMs. We therefore propose that the potential electrophysiological and pro-arrhythmic effects of *SCN10A* variants/mutations do not occur on the cardiomyocyte level, but instead are consequent to the actions of *SCN10A*/Na_V_1.8 in other cell types such as intracardiac neurons. Indeed, we and others have previously demonstrated that Na_V_1.8 is specifically expressed and functionally relevant in murine, canine and human cardiac neurons [18, 20, 21], suggesting a function of the *SCN10A* gene product for cardiac conduction via regulation of action potential firing in intracardiac neurons [18, 21, 22].

In recent years, various GWAS studies have suggested a potential modulatory effect of *SCN10A* common genetic variants on ECG parameters such as PR and QRS interval [6–10] in addition to susceptibility to AF [11–13] and Brugada syndrome [14–16]. However, it was subsequently demonstrated that the *SCN10A* variant rs6801957 (associated with QRS duration [9]) is located within a cardiac enhancer region which interacts with the promotor of *SCN5A*. As such, rs6801957 was shown to decrease *SCN5A* expression in the heart, explaining the observed associations of this *SCN10A* variant with cardiac conduction [23]. Based on these observations and our current findings, it is therefore highly likely that the *SCN10A* locus identified in various GWAS studies exerts its modulatory effects indirectly through their impact on *SCN5A* expression and/or neuronal activity, rather than through a direct effect on cardiomyocyte electrophysiology.

While our findings indicate a lack of functional relevance for Na_V_1.8 in CMs under physiological conditions, they do not rule out a potential function during pathophysiological situations. Recently, increased *SCN10A*/Na_V_1.8 expression in human ventricular tissue isolated from heart failure and hypertrophic patients as compared to non-failing and healthy myocardium, has been demonstrated [39, 40]. Na_V_1.8 inhibition with the specific blockers A-803467 and PF-01247324 decreased late *I*_Na_ magnitude, abbreviated APD and reduced cellular-spontaneous Ca^2+−^release and proarrhythmic events in human failing and hypertrophic CMs [39, 40]. Of note, in both these studies, no electrophysiological experiments were performed in non-failing and non-hypertrophic CMs, thus precluding comparison of the effects of Na_v_1.8 inhibition in human non diseased CMs under comparable experimental settings [39, 40]. A modulatory role for *SCN10A*/Na_V_1.8 has furthermore been suggested in AF, with A-803467 administration preventing AF recurrence in a fast-pacing canine model [21]. Increased late *I*_Na_ has been observed in right atrial appendage cardiomyocytes from AF patients as compared to individuals in SR [38, 41]. Whether alterations in *SCN10A*/Na_V_1.8-based contribute to this increased late *I*_Na_ in the setting of AF will require further investigation. Interestingly, injection of A-803467 into canine cardiac ganglionated plexi (GP) [22] and canine left stellate ganglion (LSG) [42] suppressed vagal-mediated AF and ischemia-induced ventricular arrhythmia, respectively, most likely by inhibiting the neuronal activity of GP and LSG. This further underlines the potential functional involvement of *SCN10A*/Na_V_1.8 in intracardiac neurons.

In conclusion, our study demonstrates the (functional) absence of *SCN10A*/Na_V_1.8-based channels in human and rabbit atrial and ventricular CMs under basal, non-remodeled conditions. We therefore propose that the association of *SCN10A* variants with cardiac electrophysiology is likely the result of indirect effects on *SCN5A* expression and/or Na_V_1.8 activity in cell types other than CMs, including (intracardiac) neurons.

## Electronic supplementary material

ESM 1 (DOCX 1.64 mb).
