# Supplementary material for: Absence of Functional Nav1.8 Channels in Non-diseased Atrial and Ventricular Cardiomyocytes
Source: Cardiovasc Drugs Ther. 2020 Jan 8;33(6):649–60. doi: 10.1007/s10557-019-06925-6 (PMC6994555; doi:10.1007/s10557-019-06925-6)
Supplement: Supplementary file 1 — (DOCX 1.64 mb). [file 10557_2019_6925_MOESM1_ESM.docx]

**Supplemental material**

**Absence of** **functional Na_v_1.8 channels in non-diseased atrial and ventricular cardiomyocytes**

Simona Casini^1*^ PhD, Gerard A. Marchal^1^ MSc, Makiri Kawasaki^1^ PhD, Fransisca A. Nariswari^1^ MSc, Vincent Portero^1^ PhD, Nicoline W. E. van den Berg^3^, Kaomei Guan^2^ PhD, Antoine H.G. Driessen^3^ MD, PhD, Marieke W. Veldkamp^1^ PhD, Isabella Mengarelli^1^ PhD, Joris R. de Groot^3^ MD, PhD, Arie O. Verkerk^1,4^ PhD, Carol Ann Remme^1^ MD, PhD

^1^Department of Experimental Cardiology, Amsterdam UMC, Meibergdreef 15, 1105 AZ Amsterdam, The Netherlands

^2^Institute of Pharmacology and Toxicology, Technische Universität Dresden, Fetscherstrasse 74, 01307 Dresden, Germany

^3^Department of Cardiology, Amsterdam UMC, Meibergdreef 15, 1105 AZ Amsterdam, The Netherlands

^4^Department of Medical Biology, Amsterdam UMC, Meibergdreef 15, 1105 AZ Amsterdam, The Netherlands

*corresponding author: s.casini@amsterdamumc.nl

**Supplemental Methods**

**Isolation of left ventricular rabbit cardiomyocytes**

Three-four months old male New Zealand White rabbits (Charles River Laboratories) were anaesthetized with 20 mg xylazine and 100 mg ketamine (intramuscularly) and heparinized with a bolus of 1000 IU heparin (intravenously). Subsequently, the animals were sacrificed, the thorax was opened and the heart was rapidly excised and immersed in ice-cold perfusion solution (composition see below). The aorta was cannulated and the heart was connected to a Langendorff system and perfused at 37 °C with a modified Tyrode’s solution containing (in mM): 128 NaCl, 4.7 KCl, 1.45 CaCl_2_, 0.6 MgCl_2_, 27 NaHCO_3_, 0.4 NaH_2_PO_4_ and 11 glucose (pH maintained at 7.4 by equilibration with a mixture of 95% O_2_ and 5% CO_2_). Left ventricular (LV) cardiomyocytes (CMs) were isolated as previously described [1] and stored at room temperature in a HEPES-buffered solution containing (in mM): 146.5 NaCl, 3.3 KHCO_3_, 1.4 KH_2_PO_4_, 1.0 NaHCO_3_, 2.0 MgCl_2_, 1.3 CaCl_2_, 17 HEPES, 11 glucose supplied with 1% fatty acid free albumin (pH 7.3; NaOH) until further use.

**Isolation of human left atrial cardiomyocytes**

Human left atrial appendages (LAAs) were obtained from patients in sinus rhythm (SR) without a history of AF undergoing cardiac surgery (coronary bypass grafting or valve surgery). Transoesophageal echocardiography (TEE) was performed during surgery to assess left atrial (or appendicular) thrombi. The LAA was excised and retrieved directly prior to aortic cross-clamping and initiation of cardioplegia, unless removal was deemed unsafe by the surgeon. Completeness of LAA excision was confirmed by TEE [2]. Part of the LAA tissue was immediately frozen in liquid nitrogen to be subsequently used for molecular analysis, while the other part was transported to the laboratory on ice and single cells were obtained by an enzymatic isolation modified from Dobrev et al. [3]. In short, fatty tissue and endocardial connective tissue were removed and the remaining tissue was cut into small cubic pieces (≈1 mm^3^), and washed three times for 10 minutes in Ca^2+^-free MOPS ((3-(N-morpholino)propanesulfonic acid) solution (37°C) containing (in mM): 100 NaCl, 10 KCl, 5 glucose, 5 MgSO_4_*6H_2_O, 50 taurine, 11 creatine, 5 MOPS (pH 7.0; NaOH). Then, the atrial pieces were incubated for 60 minutes in Ca^2+^-free solution (37°C) to which collagenase A (250 U/mL; Roche) and proteinase XXIV (3.5-7 U/mL; Sigma) were added. In the last 45 minutes, the Ca^2+^ concentration was stepwise increased by Ca^2+^ increments of 0.05 mM to a final concentration of 0.15 mM by adding CaCl_2_ (i.e. after 15, 40 and 50 minutes). Subsequently, the pieces were placed in 0.1 mM Ca^2+^ MOPS solution (37ºC) with collagenase A (310 U/mL; Roche). During this last incubation period, the tissue pieces were gently shaken and, at regular intervals of 15 minutes, the solution was microscopically examined for the presence of dissociated rod-shaped myocytes. When rod-shaped myocytes were observed (usually after 75 minutes), cells were removed every 10 minutes for 80 minutes and transferred into Ca^2+^-free MOPS solution (37°C) containing 1% of bovine serum albumin. Single cells were stored at room temperature in a modified Tyrode’s solution containing (in mM): 140 NaCl, 5.4 KCl, 0.9 CaCl_2_, 0.5 MgCl_2_, 5.5 glucose and 5.0 HEPES, pH 7.4 (NaOH) until further use.

**Differentiation of hiPSCs into cardiomyocytes**

A human induced pluripotent stem cell (hiPSC) control line (iC113) previously generated and characterized [4] was used to generate cardiomyocytes (hiPSC-CMs) by adaptation of a previously described protocol [5]. Briefly, mesoderm induction was started in undifferentiated hiPSCs, maintained in mTeSR1 medium on matrigel-coated vessels, by treatment with CHIR 99021 (1.5 µM), Activin A (20 ng/ml), BMP4 (20 ng/ml) for 1 day in the presence of RPMI 1640 medium supplemented by B27 without insulin. The same medium without factors was used to refresh the cells the day after. Inhibition of the Wnt pathway was performed on day 3 to 5 by treatment with IWP-4 (5 µM). After day 7 cells were maintained in RPMI 1640 medium and insulin-containing B27 supplement. Spontaneously beating cells were detected approximately from day 10. Differentiation was allowed to proceed up to day 20, followed by a 5 days cardiomyocytes-enrichment step consisting in partial elimination of non-cardiomyocytes by 4 mM lactate treatment in medium depleted of glucose [6]. Subsequently these cardiomyocytes were used for RT-PCR. For electrophysiological investigation, single hiPSC-CMs were obtained by incubation of adherent cultures with Tryple Select 5x for 5-10 minutes at 37°C and gentle mechanical disaggregation. The enzyme was removed by dilution with culture medium and cells were collected by centrifugation. The hiPSC-CMs were then re-suspended and seeded in the presence of RPMI 1640 medium supplemented with insulin-containing B27 on matrigel-coated glass coverslips. Medium was replaced after 24h and subsequently every 2-3 days. hiPSC-CMs were used for electrophysiological analysis 8-12 days after dissociation.

**L-type calcium current measurements**

L-type calcium current (I_CaL_) was measured in isolated LV rabbit CMs at 36°C. The pipette solution contained (in mM): 140 CsCl, 5 K_2_-ATP, 10 HEPES, 10 EGTA; pH 7.2 (CsOH). CMs were superfused with an external solution containing (in mM): 145 TEACl, 5.4 CsCl, 1.8 CaCl_2,_ 1 MgCl_2_, 5.5 glucose, 0.2 4,4'-Diisothiocyano-2,2'-stilbenedisulfonic acid (DIDS), 5 HEPES; pH 7.4 (CsOH). I_CaL_ was measured in response to depolarizing voltage steps from a holding potential of -70 mV (cycle length of 5 seconds) and it was defined as the difference between peak and steady state current (at 300 ms). Voltage dependence of activation and inactivation curves were fitted with Boltzmann function (y=[1+exp{(V-V_1/2_)/*k*}]^-1^), where V_1/2_ is the half-maximal voltage of (in)activation and *k*, the slope factor*.* Current densities were calculated by dividing current amplitude by cell membrane capacitance (Cm). Cm was determined by dividing the decay time constant of the capacitive transient in response to 5 mV hyperpolarizing steps from -40 mV, by the series resistance (Rs). Potentials were corrected for the estimated change in liquid junction potential (11 mV).


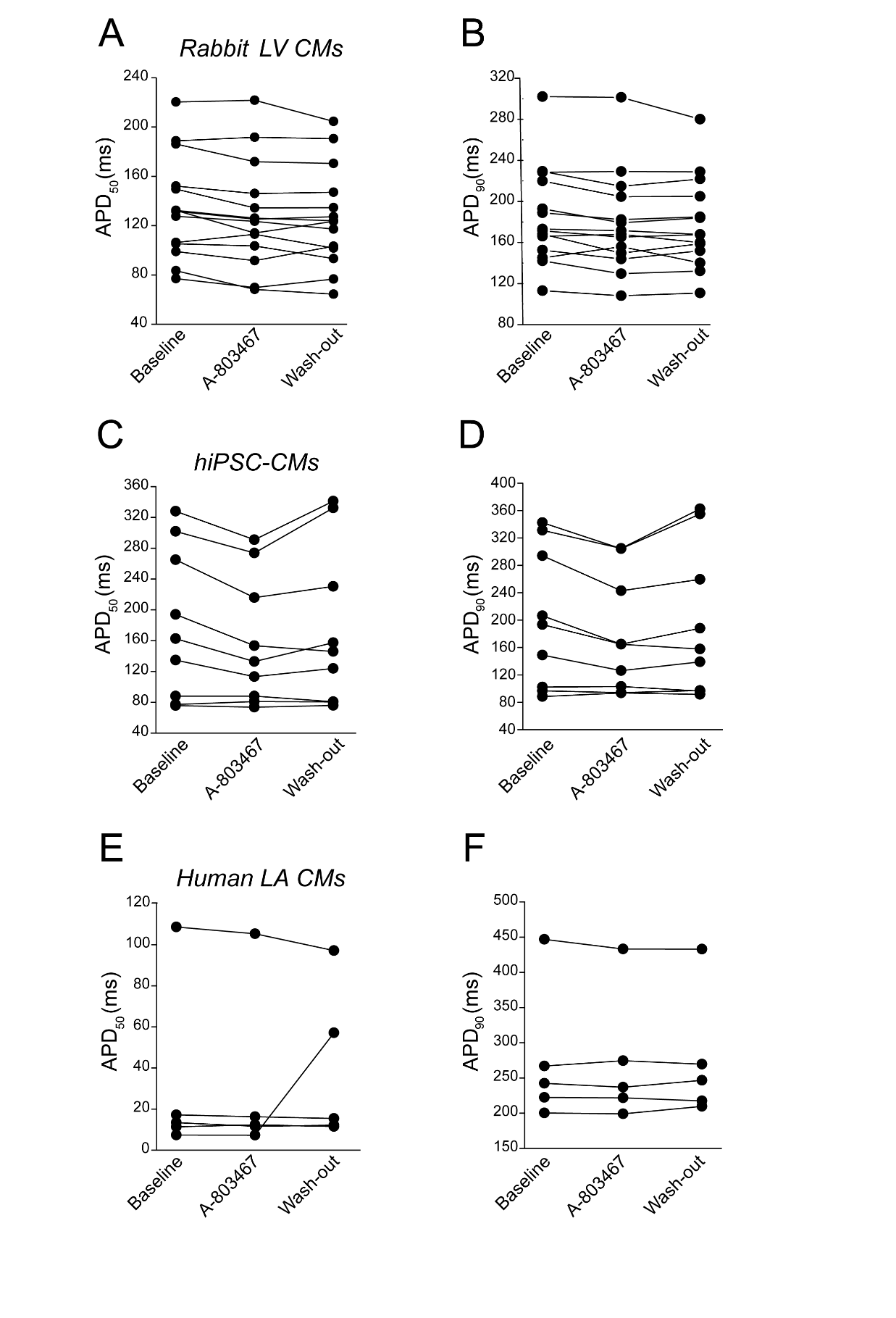


**Supplemental Figure 1.** **Scatter plots for action potential duration in atrial and ventricular cardiomyocytes.** Action potential duration (APD) values at 50% and 90% repolarization (APD_50_ and APD_90_, respectively) under physiological conditions (baseline), in the presence of 100 nM A-803467 and after wash-out of the compound, in rabbit left ventricular (LV) cardiomyocytes (CMs) (**A,B**), human-induced pluripotent stem cell derived-CMs (hiPSC-CMs) **(C,D**) and human left atrial (LA) CMs (**E,F**).

**
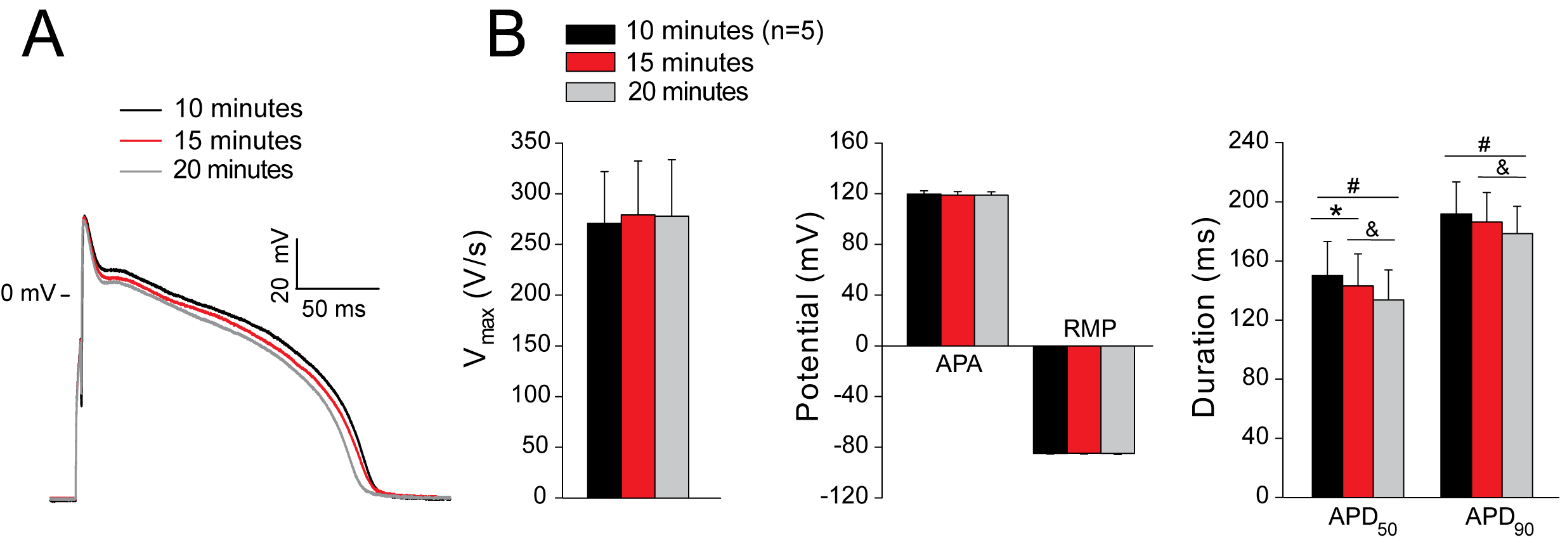
**

**Supplemental Figure 2. Vehicle-time matched experiments in left ventricular rabbit cardiomyocytes.** **A**, Examples of action potentials (APs) recorded at the stimulation frequency of 1 Hz in left ventricular (LV) rabbit cardiomyocytes (CMs) after 10, 15 and 20 minutes following seal formation, corresponding to the baseline, the time of wash-in of the Na_v_1.8 blocker, and the time of wash-out, respectively. **B**, Average data at 1 Hz for maximal upstroke velocity (V_max_), AP amplitude (APA), resting membrane potential (RMP), AP duration at 50% and 90% repolarization (APD_50_ and APD_90_) after 10, 15, 20 minutes following seal formation. n, number of CMs. *p<0.05 10 minutes v*s* 15 minutes, ^#^p<0.05 10 minutes *vs* 20 minutes, ^&^p<0.05 15 minutes *vs* 20 minutes; one way repeated measures ANOVA followed by Holm-Sidak test for post hoc analyses or one-way repeated measures ANOVA on Ranks (Friedman test) when data where not normally distributed.

**
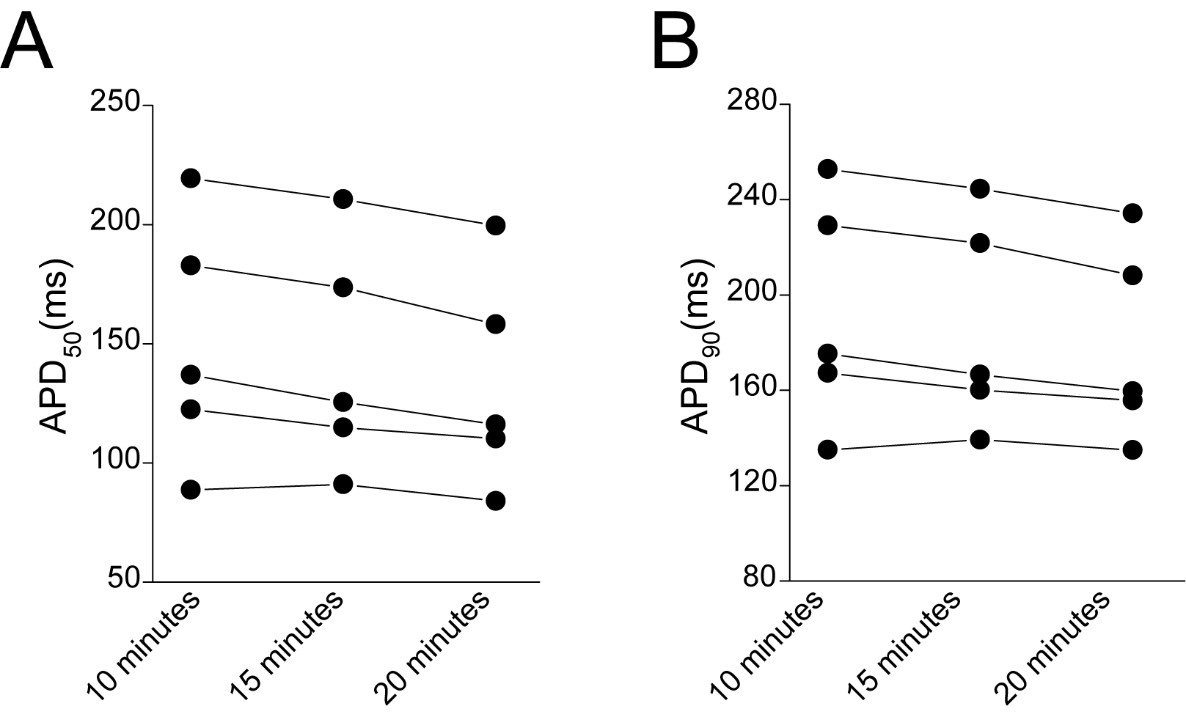
**

**Supplemental Figure 3. Scatter plots for action potential duration in left ventricular rabbit cardiomyocytes.** Action potential duration (APD) values at 50% (**A**) and 90% (**B**) repolarization (APD_50_ and APD_90_, respectively) after 10, 15 and 20 minutes following seal formation, corresponding to the baseline, the time of wash-in of the Na_v_1.8 blocker and the time of wash-out, respectively.

**
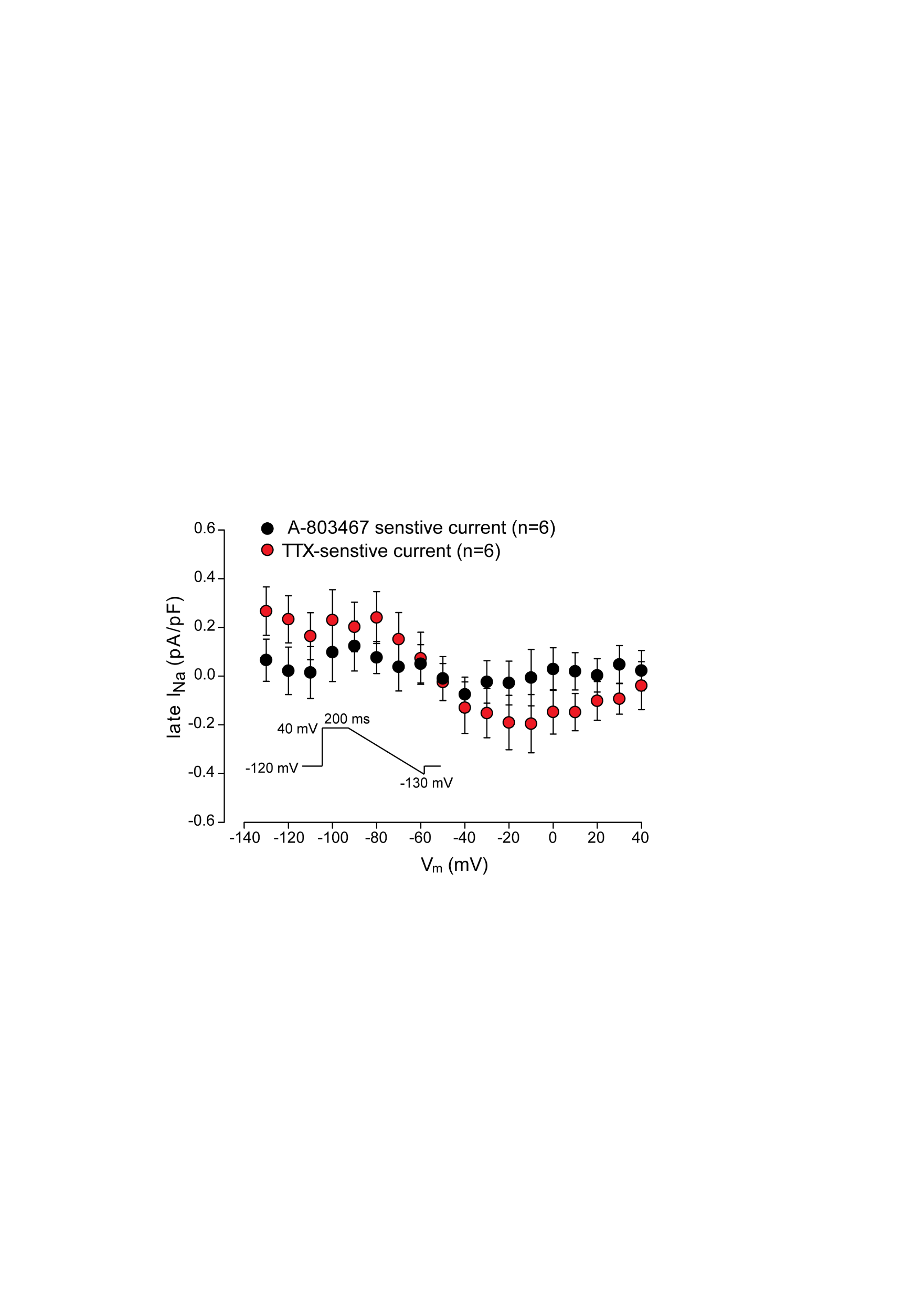
**

**Supplemental Figure 4. Na_V_1.8**-**based late sodium current (I_Na_)** **and total late I_Na_** **in rabbit cardiomyocytes.** Average current-voltage relationship in left ventricular rabbit cardiomyocytes (CMs) for Na_V_1.8**-**based late I_Na_ and total late I_Na_ measured as A-803467-and tetrodotoxin (TTX)-sensitive currents, respectively. n, number of CMs. Inset: voltage ramp protocol.


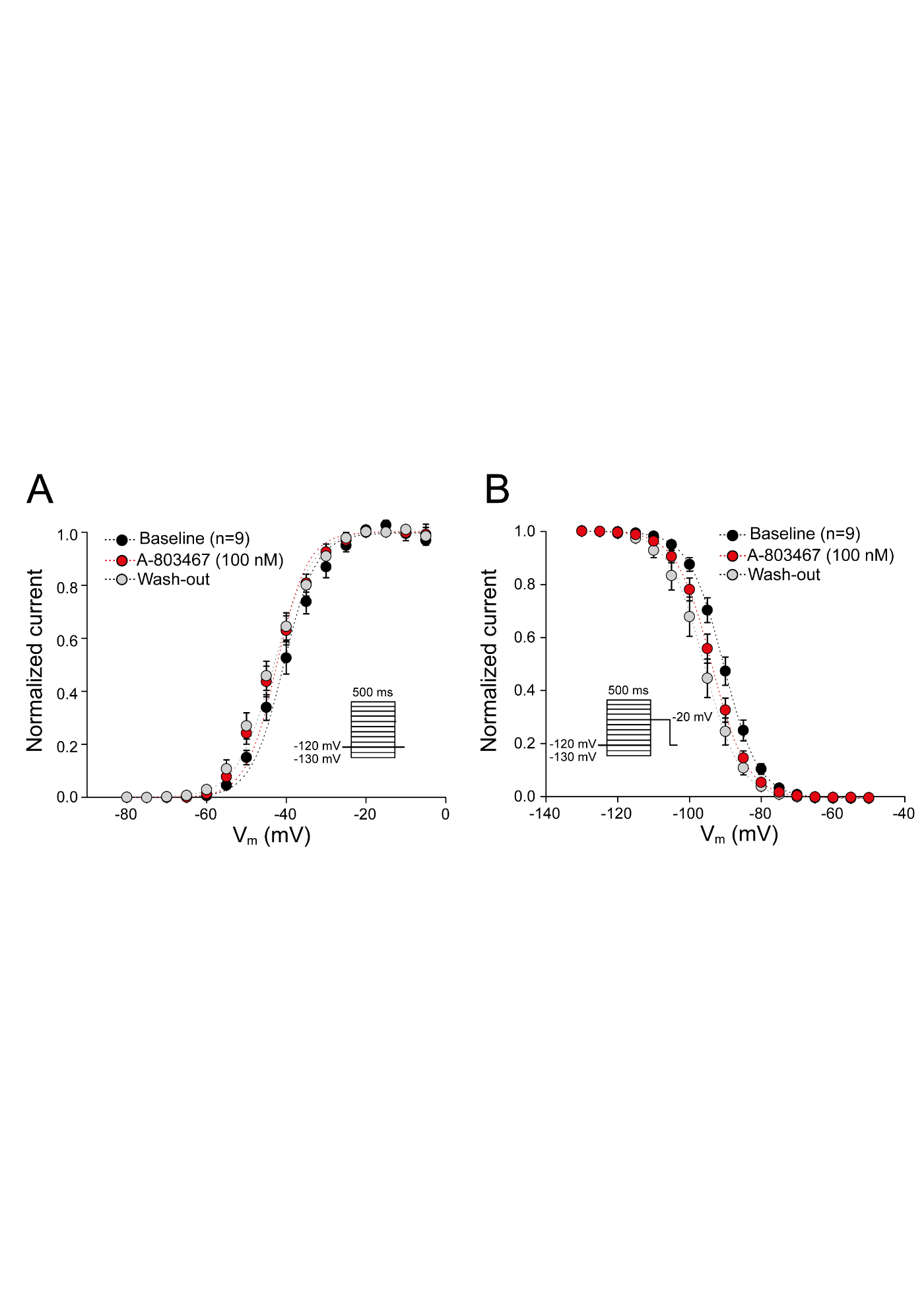
**Supplemental Figure 5. Effect of A-803467 treatment on sodium current (I_Na_) voltage dependence of activation and inactivation in human left atrial cardiomyocytes.** Average I_Na_ voltage dependence of activation (**A**) and inactivation (**B**) in human left atrial cardiomyocytes (CMs) under basal conditions, after 5 minutes wash-in of 100 nM A-803467 and 5 minutes wash-out of the compound. n, number of CMs. Insets: voltage protocols.


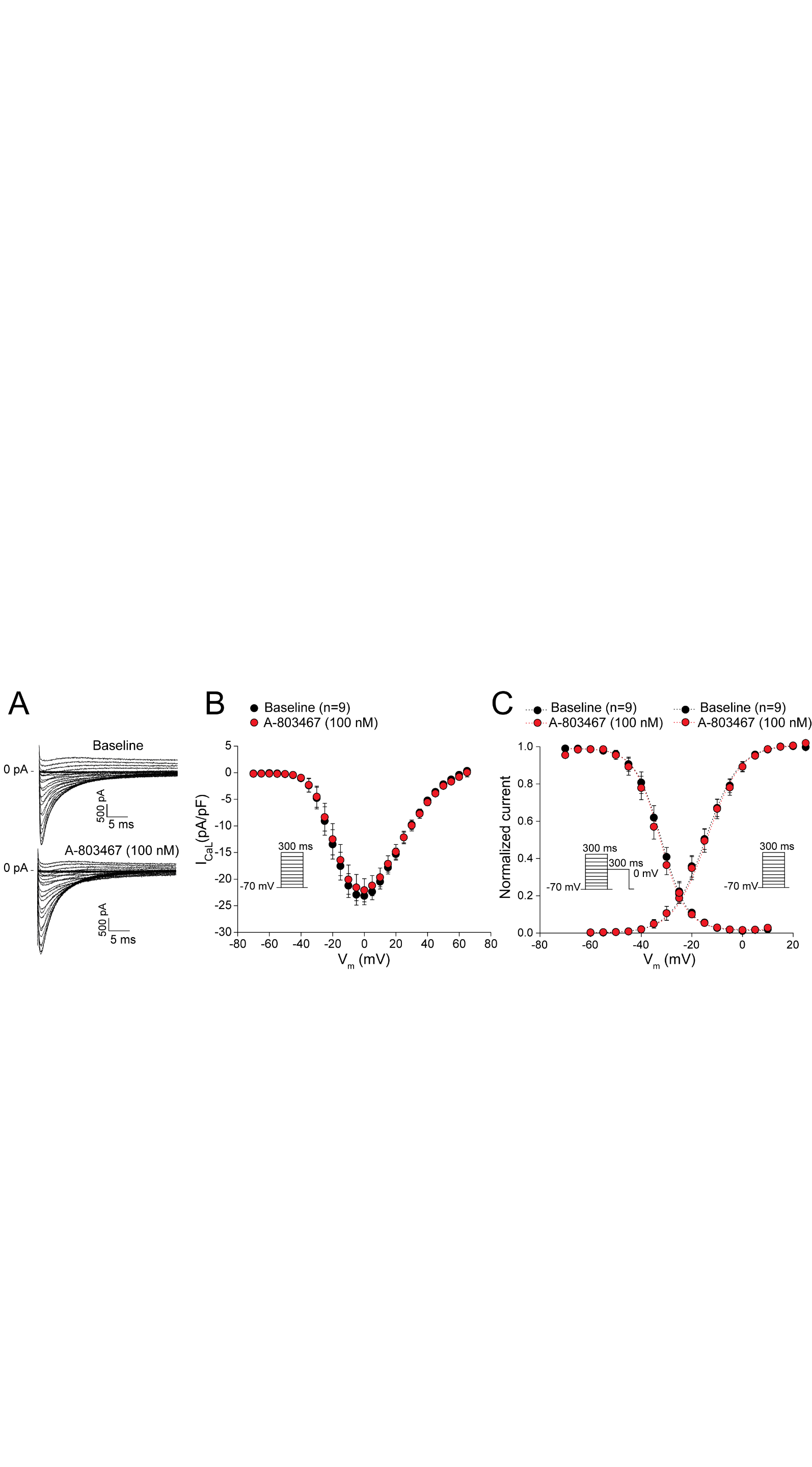


**Supplemental Figure 6. A-803467 does not affect L-type calcium current (I_CaL_) properties in rabbit cardiomyocytes.** **A**, Representative I_CaL_ traces recorded in rabbit left ventricular (LV) cardiomyocytes (CMs) under basal conditions (baseline) and after 5 minutes wash-in of 100 nM A-803467. **B**,**C**, Average current-voltage (I-V) relationship (**B**) and average I_CaL_ voltage dependence of activation and inactivation (**C**) in rabbit LV CMs at baseline and in the presence of 100 nM A-803467. n, number of CMs. Insets: voltage protocols.

**Supplemental Table 1: Clinical characteristics of patients**

|  |  |
| --- | --- |
| ***General Characteristics (n=7)*** |  |
| Gender (m/f) | 7/0 |
| Age (years) | 71.9±2.7 |
| BMI (kg/m^2^) | 27.1±1.8 |
| Heart rate (beat/min) | 77.4±4.6 |
| Hypertension (%)  Congestive HD (%)  Vascular diseases (%)  Diabetes mellitus (%) | 5 (71.4%)  0 (0%)  7 (100%)  2 (28.6%) |
|  |  |
| ***Type of surgery*** |  |
| GABG (n) | 2 |
| CABG+valve surgery (n) | 4 |
| Valve surgery (n) | 1 |
|  |  |
| ***Medication*** |  |
| Digitalis (%) | 0 (0%) |
| β-blockers (%) | 4 (57.1%) |
| ACE inhibitors (%) | 2 (28.6%) |
| Calcium channel blockers (%) | 1 (14.3%) |
| ATII blockers (%) | 2 (28.6%) |
| Antiplatelets (%) | 7 (100%) |

BMI, body mass index; CABG, coronary artery bypass grafting; congestive HD, congestive heart disease; ACE, angiotensin-converting enzyme; ATII, angiotensin II; n, number of patients. **Supplemental Table 2.** Primer sets used

|  | ***SCN5A*** | ***SCN10A*** |
| --- | --- | --- |
| hiPSC-CMs | Forward primer:   5’-GGTTGTCATCCTCTCCATCG-3’  Reverse primer:   5’-ATGCTGTTGGCGAAGGTCTG-3’ | Forward primer:  5’-GTACTACTTCACAAATGGCTGGAATG-3’  Reverse primer:  5’-GCAGGCAGGGACATCATGA-3’ |
| Human left atrial appendages | Forward primer:  5’-CATCAAGTCACTGCGGACG-3’  Reverse primer  5’-CAGACGAGGAGGACGTTCAT-3’ | Forward primer:  5’-GGAGCACAGCTGGTTTGAGA-3’  Reverse primer:  5’-TGGGCTTCTGGTCCAGGTAA-3’ |

Primer sets used for detection of *SCN5A* and *SCN10A* expression levels in human-induced pluripotent stem cell-derived cardiomyocytes (hiPSC-CMs) and human left atrial appendages.

**Supplemental Table 3.** Effect of A-803467 treatment on action potential properties in rabbit left ventricular (LV) cardiomyocytes (CMs), human-induced pluripotent stem cell derived-CMs (hiPSC-CMs) and human left atrial (LA) cardiomyocytes.

|  |  |  | **Rabbit LV CMs**  **(n=14)** | |  | **hiPSC-CMs**  **(n=9)** | | |  | **Human LA CMs**  **(n=5)** | | |
| --- | --- | --- | --- | --- | --- | --- | --- | --- | --- | --- | --- | --- |
|  |  |  | |  |  | |  |  | |  |  |  |
|  | **Baseline** | **A-803467** | | **Wash-out** | **Baseline** | | **A-803467** | **Wash-out** | | **Baseline** | **A-803467** | **Wash-out** |
|  |  |  | |  |  | |  |  | |  |  |  |
| **V_max_ (V/s)** | 213.8±14.0 | 215.8±16.1 | | 214.1±15.9 | 211.1±48.6 | | 212.3±49.2 | 205.7±48.8 | | 378.9±37.8 | 374±37.4 | 378.2±41.4 |
|  |  |  | |  |  | |  |  | |  |  |  |
| **APA (mV)** | 116.7±1.4 | 116.7±1.2 | | 116.6±1.2 | 122.4±2.1 | | 121.7±2.1 | 121.9±2.5 | | 126.5±1.1 | 126.8±1.0 | 122.1±5.1 |
|  |  |  | |  |  | |  |  | |  |  |  |
| **RMP (mV)** | -85.3±0.6 | -85.7±0.6 | | -85.8±0.6 | -84.8±0.4 | | -85.0±0.6 | -84.6±0.4 | | -75.3±3.2 | -75.8±3.3 | -74.8±3.0 |
|  |  |  | |  |  | |  |  | |  |  |  |
| **APD_50_ (ms)** | 135.2±11.1 | 128.7±11.6* | | 127.1±10.8^#^ | 180.8±32.6 | | 158.1±27.6* | 174.3±34.7 | | 31.6±19.3 | 30.6±18.8 | -38.7±16.9 |
|  |  |  | |  |  | |  |  | |  |  |  |
| **APD_90_ (ms)** | 185.2±12.7 | 178.8±12.8* | | 178.2±11.8^#^ | 200.4±33.7 | | 177.6±28.6 | 194.1±35.8 | | 275.7±44.2 | 273.1±41.9 | 275.3±40.8 |
|  |  |  | |  |  | |  |  | |  |  |  |

V_max_, maximal upstroke velocity; APA, action potential amplitude; RMP, resting membrane potential; APD_50_, APD_90_, action potential duration at 50%, 90% repolarization; n, number of CMs. *p<0.05 A-803467 *vs* baseline, ^#^p<0.05 wash-out *vs* baseline; one way repeated measures ANOVA followed by Holm-Sidak test for post hoc analyses or one-way repeated measures ANOVA on Ranks (Friedman Test) followed by Tukey test for post hoc analyses when data where not normally distributed.

**Supplemental Table 4.** Effect of A-803467 treatment on sodium current properties in rabbit left ventricular (LV) cardiomyocytes (CMs), human-induced pluripotent stem cell derived-CMs (hiPSC-CMs) and human left atrial (LA) cardiomyocytes.

|  | **Rabbit LV CMs (n=9)** | |  | **hiPSC-CMs**  **(n=7)** | |  | **Human LA CMs**  **(n=10)** | |  |
| --- | --- | --- | --- | --- | --- | --- | --- | --- | --- |
|  |  | |  |  |  | |  |  | |
|  | **Baseline** | **A-803467** | | **Baseline** | **A-803467** | | **Baseline** | **A-803467** | |
|  |  |  | |  |  | |  |  | |
| ***Current density*** |  |  | |  |  | |  |  | |
| **I_Na_ (pA/pF)** | -33.2±4.1 | -34.5±3.9 | | -56.5±8.9 | -57.6±9.1 | | -88.5±9.2 | -88.1±10.6 | |
|  |  |  | |  |  | |  |  | |
| ***Activation*** |  |  | |  |  | |  |  | |
| **V_1/2_ (mV)** | -37.9±0.8 | -38.8±0.9 | | -36.1±1.4 | -36.4±1.3 | | -40.5±1.1 | -43.3±1.2* | |
| ***k* (mV)** | 5.3±0.2 | 5.2±0.1 | | 7.0±0.3 | 7.1±0.4 | | 5.2±0.1 | 5.0±0.2 | |
|  |  |  | |  |  | |  |  | |
| ***Inactivation*** |  |  | |  |  | |  |  | |
| **V_1/2_  (mV)** | -82.5±1.1 | -83.1±1.2 | | -83.9±2.2 | -86.2±2.2 | | -90.6±0.9 | -93.4±1.0* | |
| ***k* (mV)** | -5.0±0.1 | -5.1±0.1 | | -6.8±0.2 | -6.6±0.2 | | -4.6±0.1 | -4.7±0.1 | |

I_Na_, maximal sodium current density; V_1/2_ of (in)activation, half-voltage of (in)activation; *k*, slope of the (in)activation curve; n, number of CMs. *p<0.05 A803467 *vs* baseline; paired Student’s t-test.

**Supplemental Table 5.** Effect of A-803467 treatment on L-type calcium current properties in rabbit left ventricular (LV) cardiomyocytes (CMs).

|  | **Rabbit LV CMs (n=9)** | |  |
| --- | --- | --- | --- |
|  | **Baseline** | **A-803467** | |
|  |  |  | |
| ***Current density*** |  |  | |
| **I_CaL_ (pA/pF)** | -23.1±1.7 | -22.1±2.2 | |
|  |  |  | |
| ***Activation*** |  |  | |
| **V_1/2_ (mV)** | -14.9±1.9 | -14.8±2.0 | |
| ***k* (mV)** | 6.9±0.2 | 6.8±0.2 | |
|  |  |  | |
| ***Inactivation*** |  |  | |
| **V_1/2_  (mV)** | -32.6±1.5 | -33.7±1.7 | |
| ***k* (mV)** | -4.8±0.1 | -4.7±0.1 | |

I_CaL_, maximal L-type calcium current density; V_1/2_ of (in)activation, half-voltage of (in) activation; *k*, slope of the (in)activation curve; n, number of CMs.

**Supplemental references**

1. Veldkamp MW, Geuzebroek GSC, Baartscheer A, Verkerk AO, Schumacher CA, Suarez GG et al. Neurokinin-3 receptor activation selectively prolongs atrial refractoriness by inhibition of a background K(+) channel. Nat Commun. 2018;9:4357.

2. van den Berg NWE, Neefs J, Berger WR, Boersma LVA, van Boven WJ, van Putte BP et al. PREventive left atrial appenDage resection for the predICtion of fuTure atrial fibrillation: design of the PREDICT AF study. J Cardiovasc Med (Hagerstown). 2019;20:752-61.

3. Dobrev D, Wettwer E, Himmel HM, Kortner A, Kuhlisch E, Schuler S et al. G-Protein beta(3)-subunit 825T allele is associated with enhanced human atrial inward rectifier potassium currents. Circulation. 2000;102:692-7.

4. Dudek J, Cheng IF, Balleininger M, Vaz FM, Streckfuss-Bomeke K, Hubscher D et al. Cardiolipin deficiency affects respiratory chain function and organization in an induced pluripotent stem cell model of Barth syndrome. Stem cell research. 2013;11:806-19.

5. Dambrot C, Braam SR, Tertoolen LG, Birket M, Atsma DE, Mummery CL. Serum supplemented culture medium masks hypertrophic phenotypes in human pluripotent stem cell derived cardiomyocytes. J Cell Mol Med. 2014;18:1509-18.

6. Tohyama S, Hattori F, Sano M, Hishiki T, Nagahata Y, Matsuura T et al. Distinct metabolic flow enables large-scale purification of mouse and human pluripotent stem cell-derived cardiomyocytes. Cell Stem Cell. 2013;12:127-37.
